# Recent advances in understanding colorectal cancer

**DOI:** 10.12688/f1000research.14604.1

**Published:** 2018-09-24

**Authors:** Sebastian Stintzing

**Affiliations:** 1 Department of Medicine III, University Hospital, LMU Munich, Munich, Germany

**Keywords:** colorectal cancer, BRAF, MSI-h, RAS

## Abstract

The achievements in the treatment of metastatic colorectal cancer during recent years are based on a better understanding of the disease and individualized regimen planning. In adjuvant treatment, the highly important IDEA (International Duration Evaluation of Adjuvant Chemotherapy) study has shown that treatment duration can safely be reduced in selected patient populations. In patients with pN1 and pT1-pT3 tumors, 3 months of treatment with 5-fluorouracil and oxaliplatin is comparable with respect to 3-year survival rate to 6 months of treatment. For patients with N2 tumors, 6 months of treatment should stay the standard of care. The limitation of the duration of the adjuvant treatment is significantly reducing the chemotherapy-induced morbidity. New studies will explore the use of immune-checkpoint inhibitors in the adjuvant setting in microsatellite-instable (MSI) tumors. In metastatic disease, next to the required molecular testing for *RAS* and *BRAF* mutations, MSI testing is recommended. In the rare group of patients with a MSI tumor, immune-checkpoint inhibition is changing the course of the disease dramatically. Therefore, it is important to identify those patients early. For the *RAS*-mutant cases, no new and targeted treatment options have been identified yet. An optimal treatment strategy for those patients is urgently needed. *RAS* wild-type patients with tumors derived from the left side of the colon (splenic flexure to rectum) should be treated in first line with epithelial growth factor receptor (EGFR) antibodies. This selection by a molecular and a clinical marker increased the benefit derived by EGFR antibodies dramatically and defined the most effective treatment option for those patients. New selection criteria based on gene expression, methylation, and other molecular changes are explored and will further influence our therapeutic strategies in the future.

## Introduction

During recent years, the treatment of colorectal cancer (CRC) has changed because of a better understanding of the biology of the disease and because of implementation of molecular and clinical biomarkers guiding clinical decisions. After years without any progress in the adjuvant setting, the IDEA (International Duration Evaluation of Adjuvant Chemotherapy) study collaboration challenged the duration of adjuvant treatment and encouraged clinicians to use a more individualized approach for patients with Union for International Cancer Control (UICC) stage III tumors. Data from the head-to-head trials CALGB 80405 (Alliance/SWOG) ^1^ and FIRE-3 ^2^ comparing anti-EGFR (anti-epithelial growth factor receptor) and anti-VEGF (anti-vascular endothelial growth factor) strategies in combination with a doublet chemotherapy in the first-line treatment of metastatic CRC (mCRC) gave us a better understanding of who will most likely benefit from an anti-EGFR strategy and influenced the current guidelines. In this article, recent advances in the understanding of colorectal cancer and future perspectives will be discussed on the basis of molecular and clinical data. The management of locally advanced rectal cancer is excluded from this review.

## Adjuvant treatment: advances and further perspectives

Standard adjuvant treatment with fluoropyrimidine (FP) plus oxaliplatin for a duration of 6 months has been defined for patients with UICC stage III tumors by the pivotal MOSAIC ^3^ and NSABP C-07 ^4^ trials. Via the addition of oxaliplatin to 5-fluorouracil/leucovorin (5-FU/LV), a 5-year overall survival (OS) rate gain of 2.7% was achieved at the cost of an increased neurotoxicity (grade 2+) of 30.4% versus 3.6% ^4^. For the MOSAIC trial, an increased 6-year OS rate of 2.5% was reported with a long-term neuropathy rate (any grade) of 15.4% after 48 months of follow-up for the FOLFOX-treated group ^3^. As oxaliplatin-induced neuropathy depends on the duration of oxaliplatin treatment, the question was whether it is necessary to treat UICC stage III tumors for a duration of 6 months. The IDEA collaboration prospectively pooled data from eight clinical trials conducted worldwide to answer the question of whether 3 months of adjuvant FP and oxaliplatin treatment in UICC stage III colorectal cancer is as effective as 6 months of treatment with regard to 3-year disease-free survival (DFS) ^5^. With more than 12,800 patients pooled, the trial was not able to establish non-inferiority for 3 months of treatment compared with 6 months of treatment. Both treatment arms showed appropriate efficacy with 74.6% 3-year DFS and 75.5% 3-year DFS for the respective arms (hazard ratio [HR] 1.07, 95% confidence interval [CI] 1.00–1.15). Therefore, 3 months of treatment does not equal 6 months of treatment, and the study did not meet its primary endpoint. On the other hand, toxicities, especially neuropathy, were significantly less frequent in the 3-month cohort. For neuropathy of grade 3 and higher, the frequency was 3% versus 12% in favor of the 3-month treatment cohort. So it may be argued that with an absolute difference in 3-year DFS of 0.9% and a significant difference in toxicity, we would take the risk of a higher recurrence probability to gain the significantly lower rate of toxicity. But subgroup analyses looked into populations of a higher probability of recurrence (T4 or N2 stage) or a lower risk of recurrence (T1–3, N1). In the process, it became clear that for the lower-risk group, 3 months of treatment was non-inferior to 6 months (HR 1.01, 95% CI 0.9–1.12) but that for the higher-risk group 3 months of treatment was inferior to 6 months (HR 1.12, 95% CI 1.03–1.23). This gives us the opportunity to individualize adjuvant treatment for UICC stage III tumors. Another interesting finding was the difference between capecitabine-based treatment (CAPOX) and the infusional 5-FU (FOLFOX) regimen. It appeared that 3 months of CAPOX was non-inferior to 6 months of CAPOX but that 3 months of FOLFOX was inferior to 6 months of FOLFOX. This finding raised the question of whether CAPOX is the better adjuvant treatment when compared with FOLFOX. The recommendation, therefore, is to treat patients with a low risk of recurrence (T1–3, N1 tumors) for 3 months using CAPOX, whereas patients with a high risk of recurrence should be treated for 6 months using CAPOX or FOLFOX.

For patients with UICC stage II tumors, treatment is recommended only if one of the following risk factors is revealed: T4 tumor, tumor perforation, fewer than 12 lymph nodes resected, or emergency surgery without oncological resection of the respective colon segment. For all other UICC stage II tumors, the absolute benefit of adjuvant treatment is about 2% in 3-year DFS, so adjuvant treatment is not recommended in general. In patients with high-risk UICC II tumors, no conclusive data are available to use a fluoropyrimidine/oxaliplatin combination. Subgroup analyses were not able to show a significant survival benefit for the combination treatment when compared with FP single-agent use ^6^. The 10-year DFS difference was 3.7% and was not significant for the use of oxaliplatin ^6^. Therefore, adjuvant treatment in patients with UICC stage II tumors should consist of FP monotherapy. From a molecular standpoint, there are two biomarkers that lately have drawn attention and are about to be tested in prospective trials.

CDX2 (caudal type homeobox transcription factor 2) is a protein that plays an important role during gut development and is used as a marker for gastrointestinal differentiation of adenocarcinoma. Loss of CDX2 in colon cancer can be used to define an undifferentiated carcinoma and is associated with a worse prognosis. A well-performed retrospective analysis of CDX2 showed that patients with stage II colorectal cancer benefitted from adjuvant treatment if their tumor was CDX2 negative ^7^. On the other hand, UICC stage II tumors that were CDX2 positive had a good prognosis that was not further improved by adjuvant treatment. The authors conclude that patients with stage II disease should be tested for CDX2 by immunohistochemistry and may be treated with adjuvant treatment if they bear a CDX2-negative tumor.

Another molecular marker that has stimulated phase III study designs is the phosphoinositide 3-kinase (PI3K) mutation. It is found in about 15% to 20% of CRCs and plays an important role within the EGFR-dependent pathway. In two large retrospective analyses, it has been shown that patients who received co-medication at a daily dose of 81 to 300 mg aspirin bearing a PI3K-mutant tumor had significantly longer survival in the adjuvant setting when compared with patients not taking aspirin. This led to three phase III trials prospectively investigating the use of 81 mg aspirin in the adjuvant setting of PI3K-mutant tumors: NCT02467582 (SAKK 41/13 – Aspirin), NCT00565708 (ASCOLT), and NCT02647099 (ALASCCA).

Microsatellite instable (MSI-h) tumors account for about 10% to 15% of UICC II and III tumors and are associated with a better outcome when compared with MSS (microsatellite stable) tumors. It has been known for a long time that patients with MSI-h tumors do not benefit from an adjuvant treatment using 5-FU as a monotherapy but will benefit from an adjuvant treatment using the combination of FP and oxaliplatin. The Cancer Genome Atlas (TCGA) data on colorectal cancer demonstrated the association of hypermutant tumors with MSI. Hypermutant tumors have more neo-antigens which are able to trigger an immune reaction, leading to a destruction of tumor cells. Immune-checkpoint inhibitors have proven efficacy in the treatment of MSI-h colorectal cancers and therefore are approved by the US Food and Drug Administration for the treatment of MSI-h mCRC. The ongoing ATOMIC trial by the Alliance group will prospectively investigate the use of atezolizumab, a programmed death-ligand 1 (PD-L1) inhibitor, in combination with FOLFOX versus FOLFOX alone for the treatment of stage III CRC. Hopefully, this approach will further personalize the treatment options in the adjuvant setting of CRC.

## Understanding metastatic colorectal cancer

Owing to a better understanding of the underlying carcinogenesis, the treatment of mCRC has become more diverse and more effective for specific and molecularly and clinically defined subgroups. Since 2009, the National Comprehensive Cancer Network (NCCN) guideline for the treatment of CRC has recommended *KRAS* exon 2 testing, which was expanded to *RAS* mutation testing in 2013 prior to first-line treatment in mCRC. Currently, extended *RAS* testing including the *RAS* mutations in *KRAS* exon 2, 3, and 4 and *NRAS* exon 2, 3, and 4 is recommended ^8^ to define those patients whose tumors most likely will not respond to anti-EGFR treatment. Unfortunately, only about 31% of patients with mCRC in the US are being tested prior to first-line treatment and NCCN guidelines appear not to be followed for the majority of patients ^9^.

Next to *RAS*, *BRAF* and MSI-h testing is encouraged, as MSI-h tumors can be treated with immune-checkpoint inhibitors, which are able to significantly change the course of the disease for this small fraction of 3% to 5% of patients with mCRC. Although the data supporting immune-checkpoint inhibition in MSI-h mCRC derive from a series of non-randomized trials, nivolumab ^10^ and pembrolizumab ^11^ have shown unparalleled tumor response, progression-free survival (PFS), and OS rates for patients with MSI-h tumors. The addition of the CTLA4 inhibitor ipilimumab to nivolumab led to even higher survival rates than did nivolumab alone at the price of a higher frequency of side effects ^10^. Whether there is a difference in efficacy between the two PD-1 inhibitors pembrolizumab and nivolumab has not yet been determined. MSI-h leads to hypermutant tumors which display numerous neo-antigens. Those antigens can be recognized by the immune system when PD-1/PD-L1 immune-checkpoint blockade is inhibited by either a PD-1 or a PD-L1 antibody. Next to MSI-h, polymerase epsilon ( *POLE*) mutations can result in hypermutant tumors. Within the TCGA set, roughly 25% of the hypermutant tumors (16% of the analyzed cohort) displayed a *POLE* mutation ^12^, which makes it worthy of testing for in the metastatic setting. Case reports show activity of immune-checkpoint inhibition in this special patient subgroup.

*BRAF*-mutant patients have a worse prognosis when compared with patients with all other tumors in mCRC. Median survival times in retrospective analyses do not exceed 20 months ^13^. There is an unmet medical need to improve the treatment possibilities in mCRC. In a first attempt paralleling the use of BRAF inhibitors in *BRAF*-mutant malignant melanoma, single-agent BRAF inhibition was prospectively tested in a single-arm phase II trial ^14^. Disappointingly, the response rate was low at 5% (1/21) and almost all tumors had progressed at the first follow-up. Therefore, the horizontal use of BRAF inhibition on *BRAF*-mutant tumors failed to prove futility. The functional workup of this failure revealed a feedback loop, inhibiting EGFR signaling in *BRAF*-mutant CRC cells. This led to a trial investigating the use of BRAF inhibitors in combination with EGFR inhibition. A phase II study testing cetuximab plus irinotecan with or without vemurafenib (VIC regimen) has shown encouraging results ^15^. The combination of anti-EGFR plus irinotecan with vemurafenib led to a median PFS of 4.3 months versus 2.0 months for cetuximab and irinotecan alone ( *p* = 0.001). The VIC regimen had a disease control rate (DCR) of 67% versus 22% for the control arm ( *p* = 0.001) ^15^. Therefore, the VIC regimen may be used in refractory patients with a *BRAF*-mutated tumor. Ongoing studies, such as the phase III BEACON study, test a second-line chemotherapy-free regimen using cetuximab and a BRAF inhibitor (encorafenib) with or without a MEK inhibitor (binimetinib) against FOLFIRI (5-FU, folinic acid, and irinotecan) or irinotecan plus cetuximab. Translational workup of *BRAF*-mutant mCRC cases revealed at least two different *BRAF*-mutant mCRC types ^16^: one accounting for about a quarter of the cases, in which gene expression revealed an activation of the RAS–PI3K–AKT axis of the EGFR-dependent secondary pathway (“BM1”), and a second type (“BM2”) (about three-quarters of the cases), in which BRAF–MEK–ERK and the activation of the cell cycle are paramount. Therefore, at least two different *BRAF*-mutant mCRC types have to be attributed where probably the current treatment strategies will show different efficacy. Retrospective translational workup will be vital to understanding and classifying the clinical data produced by those trials.

For *RAS*-mutant tumors, no specific treatment options are available. The prognosis remains unchanged for the last decade, with median survival times of 20 to 24 months. Standard treatment options include double chemotherapy using FOLFIRI or FOLFOX combined with bevacizumab in the first line ^17^. The addition of oxaliplatin to FOLFIRI plus backbone did not prolong survival for the *RAS*-mutant subgroup and therefore, owing to its increased toxicity and limited efficacy, this combination should not be used as first-line treatment ^13^.

The most controversially discussed data of the last few years have come from studies investigating anti-EGFR versus anti-VEGF antibody treatment in combination with chemotherapy in first-line *RAS* wild-type patients.

The EGFR antibodies cetuximab and panitumumab have been shown to significantly increase tumor response rates (objective response rate, or ORR), PFS, and OS when added to first-line chemotherapy in patients with *RAS* wild-type tumors ^18, 19^. Subsequently, both drugs have been approved for the combination with FOLFOX (5-FU, folinic acid, and oxaliplatin) or FOLFIRI or as single-agent therapy. Within the extended *RAS* wild-type population, three head-to-head trials evaluated the efficacy of first-line chemotherapy plus either bevacizumab or EGFR antibodies. The phase II PEAK trial used FOLFOX backbone chemotherapy and compared bevacizumab with panitumumab, and PFS was the primary endpoint ^20^. FIRE-3 (AIO KRK-0306) combined FOLFIRI and bevacizumab or cetuximab and, in a phase III setting, evaluated tumor response rate (ORR) as the primary endpoint ^2^. The CALGB/SWOG (Alliance) 80504 study was a phase III trial comparing cetuximab with bevacizumab with an FP- and irinotecan- or oxaliplatin-containing regimen, and OS was the primary endpoint ^1^. In CALGB 80405, the choice of the backbone chemotherapeutic regimen was at the physician’s discretion. Meta-analyses of those three trials showed significantly higher response rates (odds ratio of 0.57) and a significantly longer OS (HR 0.8) for the anti-EGFR combinations when compared with bevacizumab combinations ^21, 22^. With respect to toxicities, all three trials showed comparable toxicities for both the anti-EGFR and the bevacizumab combinations. Quality of life has been measured in the CALGB 80405 study using the QLQ-C30 questionnaire and showed no difference between cetuximab- or bevacizumab-treated patients ^23^. Only the skin satisfaction evaluation showed differences for the first 2 months of treatment favoring bevacizumab-treated patients ^23^.

The retrospective analyses of numerous trials have revealed a strong prognostic effect of the primary tumor location on survival irrespective of the chemotherapy used, stage of the disease, or other known prognostic factors ^24– 26^. Data have become more intriguing since the studies using EGFR antibodies have been analyzed ^24^. Right-sided tumors derive from the cecum, ascending colon, and transverse colon, whereas left-sided tumors originate from the descending colon, sigma, and rectum ^27^. Within the CRYSTAL trial, the addition of cetuximab to first-line FOLFIRI did not affect OS in patients with right-sided *RAS* wild-type tumors, but a significant and clinically relevant OS benefit of a median of about 7 months could be established (HR 0.65, *p* = 0.002) ^24^. Similar results could be seen for the FIRE-3 trial where in left-sided tumors the median OS was 10 months longer for the FOLFIRI cetuximab-treated patients when compared with the FOLFIRI bevacizumab-treated patients (HR 0.63, *p* = 0.002) ^24^. Two meta-analyses using the available data on sidedness for panitumumab-, cetuximab-, and bevacizumab-based trials showed that, for *RAS* wild-type tumors, left-sided primaries benefit with regard to ORR, PFS, and OS when treated in first line with EGFR antibodies ^26, 28^. The clinical importance of the primary tumor location is reflected in the current NCCN ^29^, European Society for Medical Oncology (ESMO), and ESMO-Asia ^30^ guidelines. For first-line treatment of mCRC, ESMO guidelines recommend EGFR antibodies in combination with chemotherapy for patients with left-sided, *RAS* wild-type primaries. NCCN guidelines recommend both EGFR and VEGF antibodies in left-sided *RAS* wild-type primaries. For right-sided tumors and *RAS*-mutant tumors, chemotherapeutic combinations with VEGF antibodies are favored in both guidelines.

Biological reasons for those clinical differences are manifold and have not yet been fully explained. The molecular makeup of right-sided tumors shows a higher frequency of *BRAF* mutations and MSI-h tumors, whereas *RAS* mutations seem to be equally distributed between right- and left-sided tumors ^31^. The CpG-island methylated phenotype (CIMP) is more common in right-sided tumors ^32^, and hypermethylation has also been defined as a negative predictive biomarker for the use of EGFR antibodies ^33^. Interestingly, even after adjustment for the known prognostic factors of mCRC, including age, gender, and mutations, sidedness remained an independent prognosticator ^34^ for OS.

In addition to the known and clinically relevant mutations of biomarkers *BRAF* and *RAS* and MSI-h, gene expression analyses have led to a new classification of colorectal cancer. Using data from more than 3,400 CRC cases (most of which were UICC stage II and III), the consensus molecular subgroup (CMS) consortium has revealed at least four different types of CRC ^35^. Those subgroups are of prognostic value and are defined by recurrent gene expression patterns. CMS1 is the immunological subtype which includes MSI-h tumors, whereas CMS2 has a biology reflecting the classic adenoma-carcinoma sequence with WNT-pathway alterations and EGFR-pathway activation. CMS3 is characterized by metabolic dysregulation such as an elevated glutaminolysis and differs vastly from CMS4, which is defined by an upregulation of EMT (epithelial-mesenchymal transition) and transforming growth factor-beta (TGF-β). The prognostic value could be validated using data of two phase III studies ^36, 37^. Interestingly, no clear predictive value could be revealed for the use of either cetuximab or bevacizumab. The question is whether the use of oxaliplatin or irinotecan (or both) can be defined by those subgroups. More importantly, these subgroups may help us to obtain a better understanding of the dysregulation that leads to carcinogenesis in mCRC and eventually will help us to define new targets to revolutionize trial designs and treatment for mCRC.

In further line treatment of *RAS* wild-type tumors, HER2-targeted therapy becomes another option to personalize mCRC treatment. The phase II HERACLES trial investigated ORR of lapatinib plus trastuzumab in patients with treatment-refractory *KRAS* codon 12/13 wild-type, HER2-positive mCRC ^38^. Within this group of chemorefractory patients, 30% (n = 8) of patients achieved an objective response and 44% (n = 12) had stable disease. Given that only 22% (n = 6) of patients experienced toxicity of grade 3 or higher, the combination of lapatinib plus trastuzumab is an option for chemorefractory patients. This may be of special importance for EGFR antibody-pretreated patients, as HER2 upregulation is a known mechanism of secondary resistance against anti-EGFR substances. Unfortunately, HER2 overexpression remains a rare event in mCRC with a frequency of about 5.5% of all mCRC cases ^38^.

## Conclusions

Colorectal cancer is not one disease but many. For today’s clinical practice, testing for mutations in *RAS* and *BRAF* genes as far as the analysis of the MSI status is imperative to provide the best possible treatment to the individual patient. Personalized medicine includes the use of different first-line chemotherapy regimens and a meaningful use of antibodies. There is no one-size-fits-all regimen anymore. Differences seen in the clinical outcome of right- and left-sided primaries stimulated further investigations, including methylation, gene mutational, and gene expression analyses. Those gave us deeper insights into the molecular makeup of mCRCs and eventually will lead to new and more effective treatments for mCRC.
